# *κ* Opioid Receptor Agonist Inhibits Myocardial Injury in Heart Failure Rats through Activating Nrf2/HO-1 Pathway and Regulating Ca^2+^-SERCA2a

**DOI:** 10.1155/2021/7328437

**Published:** 2021-07-30

**Authors:** Tengfei Wu, Hui Yao, Binghua Zhang, Shenglai Zhou, Ping Hou, Keyan Chen

**Affiliations:** ^1^Department of Laboratory Animal Science, China Medical University, Shenyang, Liaoning 110122, China; ^2^Department of Congenital Heart Disease, General Hospital of Northern Theater Command, Shenyang, Liaoning Province 110016, China; ^3^Sino-British Union College, China Medical University, Shenyang, Liaoning 110122, China; ^4^Department of Cardiology, Liaoning University of Traditional Chinese Medicine, Shenyang, Liaoning 110032, China

## Abstract

**Objectives:**

We aimed to observe the protective effect of *κ* opioid receptor (*κ*-OR) agonist on myocardial injury in heart failure (HF) rats and its effect on Ca^2+^-SERCA2a and to explore the regulatory mechanism with the Nrf2/HO-1 signaling pathway.

**Methods:**

50 Sprague-Dawley rats were randomly divided into the following groups: the sham operation group (sham group), HF model group (HF group), HF+*κ*-OR agonist U50488 group (HU group), HF+U50488H+novel calmodulin-dependent protein kinase II (CaMKII) agonist (oleic acid) (HUO group), and HF+U50488H+Nrf2 inhibitor (HUM group). The HF rat's model was established through surgical ligation of the left anterior descending coronary artery and the exhausting swimming exercise. After that, rat's cardiac function was monitored by echocardiography. HE and MASSON staining was used to detect the myocardial injury, and TUNEL staining was used to detect the myocardial apoptosis. ELISA was performed to detect the biomarkers of oxidative stress. Moreover, the distribution of reactive oxygen species (ROS) and Nrf2 was detected under immunofluorescence. The expression of sarco/endoplasmic reticulum calcium (Ca^2+^) ATPase (SERCA) 2a, calmodulin, endoplasmic reticulum stress- (ERS-) related proteins, and Nrf2/HO-1 signaling pathway-related proteins were detected by Western Blotting.

**Results:**

*κ*-OR agonist U50488H can significantly enhance rat's cardiac function, reduce the injury and apoptosis of myocardial cells, and alleviate endoplasmic reticulum stress injury in HF rats via upregulating the SERCA2a expression and inhibiting the Ca^2+^ influx. Furthermore, U50488H could also inhibit the phosphorylation of CaMKII and cAMP-response element binding protein (CREB). Additionally, administration of CaMKII-specific agonist could partially block the therapeutic effect of *κ*-OR agonist on the myocardium of HF rats. Interestingly, the antagonist of Nrf2 could also significantly reverse the therapeutic effect of *κ*-OR agonist. Therefore, these results suggested that the effect of U50488H on HF rats is dependent on regulating CaMKII phosphorylation and activating the Nrf2/HO-1 pathway.

**Conclusion:**

*κ*-OR agonists U50488H can improve ERS in cardiomyocytes and relieve myocardial injury in HF rats through activating the Nrf2/HO-1 pathway and regulating Ca^2+^-SERCA2a to inhibit Ca^2+^ influx.

## 1. Introduction

Heart failure (HF) is caused by structural or functional abnormality of the heart, leading to impaired cardiac blood circulation, reduced cardiac function, and failure to meet the metabolic needs of the body [1]. HF was usually accompanied by pathological changes of circulation and/or pulmonary circulation congestion [2, 3]. Investigating the effective prevention and treatment measures is significant to relieve HF symptoms, reduce all-cause mortality, and enhance the quality of patients' life.

The endoplasmic reticulum (ER) is an important machine for protein processing, including protein folding and transportation [4]. The homeostasis of ER could be broken with the occurrence of ischemia [5], hypoxia [6], lipid overload [7], viral infection [8], and drugs/toxin effect [9], leading to the accumulation of folding or unfolding protein in the cavity or the imbalance state of Ca^2+^ in the ER, called endoplasmic reticulum stress (ERS) [10, 11]. As ERS response stayed for a long time, cell apoptosis was induced, and cell death happened [12]. The sarcoplasmic/endoplasmic reticulum Ca^2+^ ATPase-2a (SERCA2a) regulates the contractile and diastolic functions of myocardial cells via participating in the transport of Ca^2+^ and maintains the steady state of Ca^2+^ [13]. Decreased SERCA2a during ERS can cause the imbalance of Ca^2+^ homeostasis in myocardial cells, which induces cardiac contractile dysfunction leading to HF [14].

In the study of HF, the nuclear factor erythroid 2-related factor 2/heme oxygenase-1 (Nrf2/HO-1) signaling pathway has been demonstrated to play an essential role in improving cardiac functions and myocardial remodeling [15, 16]. The cysteine sites (Cys-273, Cys-288) on Kelch-like ECH-associated protein 1 (Keap1) were modified simultaneously during ERS, reducing the ubiquitination of Nrf2 and increasing the level of full function of Nrf2 [17]. The increased Nrf2 could upregulate the expression of HO-1 and improve the antioxidant function of cardiomyocytes through combining with angiotensin II receptor blocker (ARB) as Nrf2 entering cell nuclear, suggesting that Nrf2 participates in the processes of inhibiting cardiomyocyte apoptosis and ventricular remodelling [18, 19] [20]. Therefore, it has great clinical significance that inhibition of the ERS response of myocardial cells and maintaining the homeostasis of myocardial calcium could improve the patient's cardiac functions and reduce the hospitalization rate and mortality in HF disease.

*κ* opioid receptors (*κ*-OR) are primarily distributed in the heart [21]. The treatment of non-selective opioid receptor inhibitor cannot cause significant changes in cardiovascular activity at normal condition; however, the endogenous opioid peptide system could be activated in a stress state to increase the plasmatic opioid peptide content [22]. Moreover, the combination with opioid receptors exerts a regulatory effect on vascular function [23, 24]. Activating *κ*-OR was reported to reduce the area of myocardial infarction and to relieve arrhythmia caused by ischemia-reperfusion injury, inhibiting myocardial cell apoptosis and promoting cardiac functions, which indicated that the function of *κ*-OR is correlated with regulation of Ca^2+^ level [25, 26]. This study is aimed at observing the therapeutic effect of *κ*-OR agonist U50488H on cardiac functions and ERS response in HF rats and to investigate the regulatory mechanisms of the Nrf2/HO-1 signaling pathway to provide theoretical basis for the clinical drug development in HF.

## 2. Materials and Methods

### 2.1. Experimental Animals and Grouping

50 SPF male Sprague-Dawley rats, weighing 260-300 g, were purchased from Beijing Vital River Laboratory Animal Technology Co., Ltd. and randomly divided into five groups: the sham operation group (sham group, *n* = 10), HF model group (HF group, *n* = 10), HF+*κ*-OR agonist U50488 group (HU group, *n* = 10), HF+U50488H+specific CaMKII agonist (Oleic acid) (HUO group, *n* = 10), and HF+U50488H+Nrf2 inhibitor (ML385, HY-100523, MCE, USA) (HUM group, *n* = 10). This study was approved by the Experimental Animal Welfare and Ethics Committee of China Medical University (IACUC No. 2019104).

### 2.2. Preparation of Rat's HF Model

Rat's HF model was established successfully by the following steps. Specifically, rats were anesthetized with the intraperitoneal injection of 2% pentobarbital sodium (35 mg/kg) and then connected to a ventilator following with endotracheal intubation. A lateral incision was cut about 2 cm in length from the 0.5 cm left side of chest midline to the edge of the flat axillary margin; then, the rat's heart was exposed. Rats were endured with anterior descending artery ligation, as the electrocardiogram showed the elevation of ST segment with R wave peak and the myocardium below the ligation site changing from red to pale, indicating surgical ligation was successful. Rats were intraperitoneally injected with penicillin for 15 days. After wound healing, rats were performed to swim in water at 30 ± 1°C for 30 min/d for 15 days to establish the HF rat's model [27, 28]. The forced swimming was performed in a tank (60 cm × 100 cm × 60 cm), filled with warm water to 30 cm depth and maintained at a temperature of 30 ± 1°C. The swimming time to exhaustion was used as the index of the forced swimming capacity. The rats were assessed to be exhausted when they failed to rise to the surface of the water to breathe within 7 s [29]. The rats in the sham group were treated with antibiotics after sham operation for 15 days. After the surgical ligation of the left anterior descending coronary artery, the rats in the HU group were treated with U50488H (1.5 mg/kg) by tail vein injection every day before swimming. Besides, rats in the HUO group were cotreated with the same dose (1.5 mg/kg) of U50488H and the specific CaMKII agonist (Oleic acid) was injected via tail vein after HF modelling for 15 days. In addition, rats in the HUM group were injected intraperitoneally with ML385 (30 mg/kg) before the injection of U50488H for 15 days.

### 2.3. Cardiac Function Examination

Cardiac ultrasound was performed after treatment for 15 days. Image acquisition by M-mode echocardiography examination on the left ventricular short-axis papillary muscle plane was used to measure the left ventricular anterior wall (LVAW) and left ventricular posterior wall (LVPW) thickness and left ventricular end-diastolic (LVDd) and left ventricular end-systolic (LVDs) diameter. Then, the left ventricular ejection fraction (LVEF) and left ventricular short-axis shortening rate (LVFS) were calculated based on the data obtained previously.

### 2.4. Haematoxylin-Eosin Staining

The formalin-fixed sections of the heart tissue were gradually dehydrated in 70%, 80%, 90%, 95%, and 100% ethanol, hyalinized by xylene for more than 30 min, and embedded in paraffin. Then, the paraffin-blocked tissues were sliced into 4 *μ*m sections. After that, the deparaffinised slices were stained in haematoxylin for 5 min, washed with PBS buffer, and followed by differentiation with 1% hydrochloric acid alcohol. Straight afterwards, eosin-stained solution was performed for 30 seconds. Finally, sections were dehydrated with gradient ethanol, transparentized with xylene, and sealed with neutral resins for acceptable visualization of myocardial pathological changes with light microscope.

### 2.5. Masson Staining

Paraffin-embedded heart tissue slices were deparaffinized and rehydrated with 100%, 95%, and 70% ethanol, followed by stained by Regaud's haematoxylin method and rinsed thoroughly in distilled water. Then, slices were stained in Masson ponceau-acid solution for 10 min, then washed with 2% and differentiated with 1% molybdenum phosphate acid for 5 min. Then, the sections were directly transferred to aniline blue solution and stained for 5 min; afterwards, sections were rinsed briefly in distilled water and differentiated in 0.2% glacial acetic acid. For poststaining procedures, sections were dehydrated 95% and 100% ethanol, cleaned with xylene, and mounted in neutral resins. Visualizations of sections were then observed under light microscope.

### 2.6. TUNEL Staining

Myocardial apoptosis was determined using *in situ* cell apoptosis TUNEL assay kit (Roche Diagnostics, Mannheim, Germany). Instructions were strictly followed. The 5 *μ*m sections of paraffin- embedded myocardial tissue were deparaffinised in xylene and hydrated with 100% ethanol and 95% ethanol. Proteinase K digestion method was used as pretreatment. After rinsed in PBS-Tween 20, sections were treated with 50 *μ*l TUNEL working solution and incubated in humidified chamber at 37°C for 1 hour avoiding from light. The sections were then washed 3 times with PBS buffer and incubated with 50 *μ*l of streptavidin-HRP working solution in dark chamber for 30 min, followed by thorough flushing with PBS-Tween 20. Finally, DAPI for nuclear fluorescence staining was added to sections, fluorescence microscope was used for visualized observations and the cell apoptosis rate was calculated.

### 2.7. Immunofluorescence (IF)

Postdeparaffinized and rehydrated heart tissue sections were immersed in 3% hydrogen peroxide solution, washed with PBS buffer, and immersed in 0.1 M sodium citrate solution for antigen retrieval. Then, the sections were achieved by incubating in 10% goat serum at 37°C for 30 min. After removal of serum, reactive oxygen species (ROS) probe and Nrf2 antibody were added on the sections and incubated overnight at 4°C. Then, the sections were rinsed with 1 × PBS for 2 min/time for 3 times. Afterwards, a certain concentration of fluorescent dye-labelled secondary antibody was added to the sections and the sections were incubated at 37°C for 30 min avoiding from light, following with another 10 min incubation with DAPI at room temperature was performed for cell nuclear staining. Finally, the sections were mounted in an antifade mounting medium. Visualizations of target antigen were accessible under the fluorescence microscope observation.

### 2.8. Enzyme-Linked Immunosorbent Assay (ELISA)

The natriuretic peptide contents of brain natriuretic peptide (BNP) (CEA541Ra, USCN, China) and N-terminal probrain natriuretic peptide (NT-proBNP) (CEA485Ra, USCN, China) in rats' serum were detected by ELISA. Additionally, the levels of oxidative stress indicators superoxide dismutase (SOD) (SES134Ra, USCN, China), malondialdehyde (MDA) (CEA597Ge, USCN, China), and catalase (CAT) (SEC418Ra, USCN, China) were quantified with ELISA kits, respectively, according to the protocols. The values of absorbance at 450 nm were detected with a microplate reader, and sample contents were calculated.

### 2.9. Western Blotting

Cryopreserved rat's myocardial tissues were lysed with RIPA lysis buffer. After homogenization and centrifugation for 15 min at 12,000 rpm at 4°C, the supernatants were kept at low temperature to avoid from proteolysis. The BCA protein assay was performed for quantitation of total protein in the myocardial samples. Protein samples were loaded in SDS-PAGEs for electrophoresis, followed by membrane transfer and blocking in skim milk for two hours at room temperature. Blocked membranes were incubated overnight at 4°C with primary antibodies against lymphoma-2 (Bcl-2) (ab59348, Abcam, USA), Bcl-2 associated X protein (Bax) (ab32503, Abcam, USA), CaMKII (ab22609, Abcam, USA), p-CaMKII (ab32678, Abcam, USA), cAMP response element- binding (CREB) (ab32515, Abcam, USA), p-CREB (ab32096, Abcam, USA), SERCA2a (ab2861, Abcam, USA), dihydropyridine receptor (DHPR) (ab232983, Abcam, USA), Calcium channel voltage-dependent L-type alpha 1C subunit (Cav1.2) (ab84814, Abcam, USA), Glucose-regulated protein 78 (GRP78) (ab21685, Abcam, USA), Caspase-12 (ab62484, Abcam, USA), Nrf2 (ab89443, Abcam, USA), HO-1 (ab13243, Abcam, USA), thioredoxin (Trx-1) (ab86255, Abcam, USA), and GAPDH (ab181602, Abcam, USA) as the internal control. After PVDF membrane incubated for 1 h and washed in PBS, HRP-conjugated goat anti-rabbit secondary antibody (ab6721, Abcam, USA) was added and incubated for 1 h. After thorough rinse with PBS and TBS-Tween 20, respectively, the protein bands were applied with SuperSignal™ West Pico PLUS Chemiluminescent Substrate (CAT#34580, Thermo Fisher Scientific, USA) and analyzed using ChemiDoc MP Imaging System (Bio-Rad Laboratories, USA).

### 2.10. Statistical Analysis

The data were analyzed using SPSS 21.0 (IBM SPSS® Statistics, USA) and represented as mean ± standard deviation (S.D). Statistical analysis was performed using one-way analysis of variance (ANOVA) among/between groups following with Fisher's least significant difference (LSD) in pairwise comparison and Wilcoxon rank-sum test in groups of nonparametric/abnormal distributed.

## 3. Results

### 3.1. The Protective Effects of *κ*-OR Agonist on HF Rats

**Figure 1:**
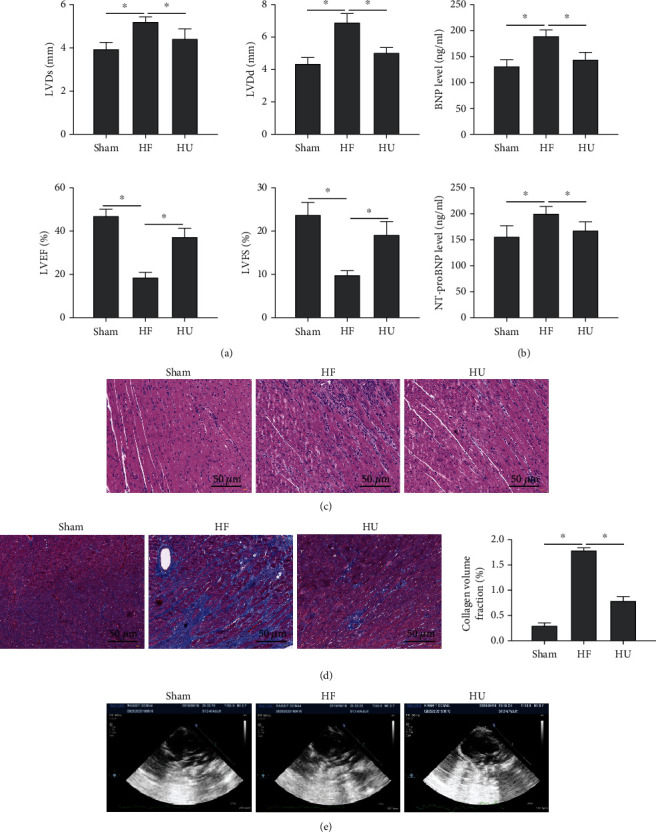
*κ*-OR agonist improved cardiac function in HF rats and reduced myocardial injury. (a) Echocardiographic parameters: LVDs, LVDd, LVEF, and LVFS. (b) The natriuretic peptide contents of BNP and NT-proBNP detected by ELISA. (c) Pathological changes observed by HE staining (scale bar = 50 *μ*m). (d) Pathological examination of tissue fibrosis by Masson staining (scale bar = 50 *μ*m); ^∗^*p* < 0.05.

To investigate the effects of *κ*-OR agonist on the HF rat's heart, we detected the left ventricular systolic function and diastolic dysfunction and the primary clinical manifestation of rats. As the HF rat model was established successfully, LVDs and LVDd diameters were notably elevated, meanwhile LVEF and LVFS were both withdrawn in the HF group (Figure 1(a), *P* < 0.05); the increased levels of BNP and NT-proBNP contents in plasma were demonstrated (Figure 1(b), *P* < 0.05), histopathology showed myocardial cell disorder and myocardial fiber rupture (Figure 1(c), *P* < 0.05), and collagen fibrin deposition leading to an increased myocardial fibrosis was observed (Figure 1(d), *P* < 0.05). These results demonstrated that highly significant correlations with the clinical indications of HF, suggesting the rat's HF modelling was established successfully (Figure 1(e)). In the HU group, the relevant indicators were all rescued with the administration of *κ*-OR agonist U50488H, implying that *κ*-OR agonist has the capability in improving myocardial injury in HF rats and enhancing the left ventricular systolic and diastolic function.

### 3.2. Pathological Enhancements by Applying *κ*-OR Agonist in HF Rats

**Figure 2:**
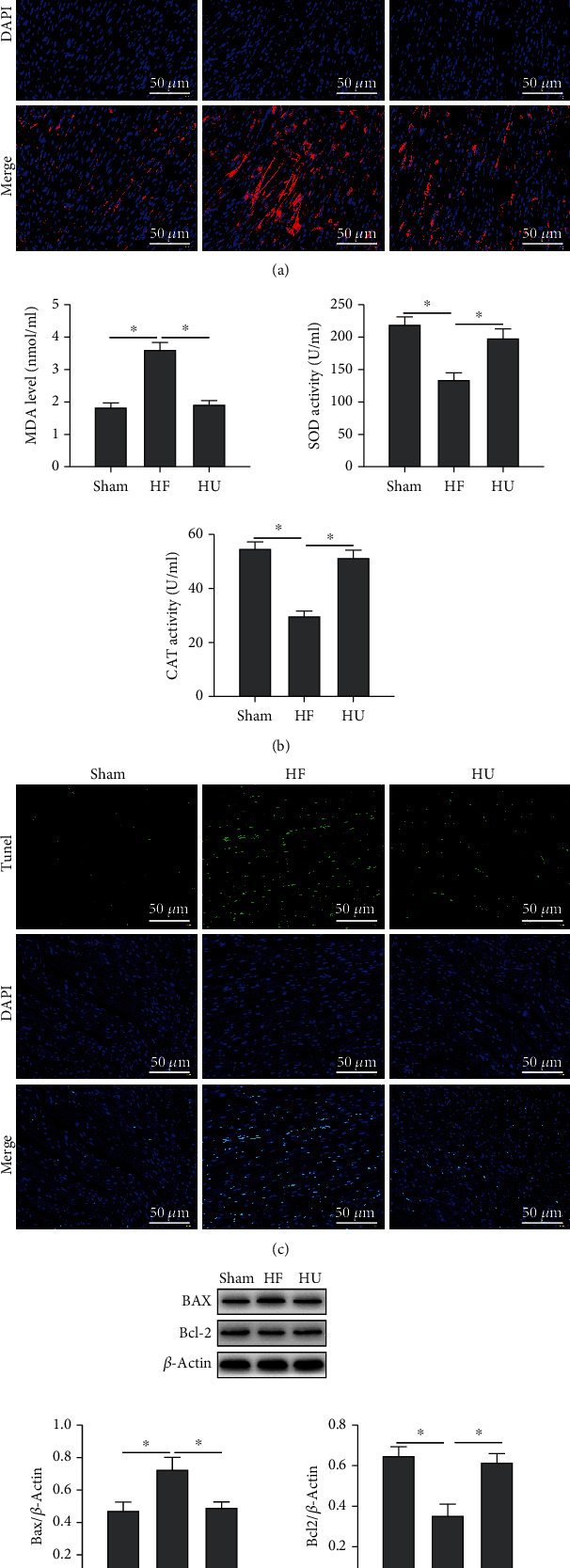
*κ*-OR agonist inhibited oxidative stress injury and reduced cardiomyocyte apoptosis in HF rats. (a) The expression of ROS in rats detected by IF (scale bar = 50 *μ*m). (b) Contents of oxidative stress factors (MDA, SOD, and CAT) in plasma detected by ELISA. (c) The rat myocardial apoptosis rate detected by TUNEL staining (scale bar = 50 *μ*m). (d) The expression levels of apoptosis-related proteins, Bax and Bcl-2, determined by Western Blotting; ^∗^*p* < 0.05.

Oxidative stress injury is one of the main causes of myocardial injury. The results showed that ROS was accumulated as HF occurs in rats (Figure 2(a)); however, *κ*-OR agonist U50488H can inhibit the release of ROS, reduce MDA contents in plasma, and promote the release of SOD and CAT (Figure 2(b)). Besides, oxidative stress is the leading cause of myocardial cell apoptosis which is the main pathological factor that accelerates the progression of HF. A significant increase in myocardial apoptosis rate in the HF group was observed (Figure 2(c)), and the reduced expression level of the apoptosis-activating factor Bax was detected with the occurrence of HF; in contrast, the expression of the antiapoptotic factor Bcl-2 was suppressed (Figure 2(d)). These pathological changes were relieved after U50488H was applied on HF rats, suggesting that *κ*-OR agonist can inhibit oxidative stress injury in HF rats and reduce cardiomyocyte apoptosis (Figure 2).

### 3.3. Regulations of *κ*-OR Agonist on ERS Homeostasis

**Figure 3:**
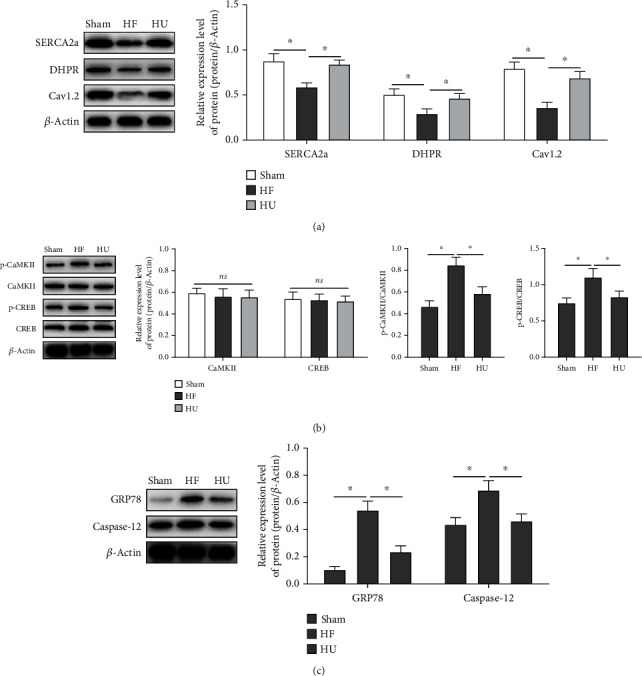
*κ*-OR agonist activates Ca^2+^-SERCA2a to inhibit ERS response in HF rats. The expression of SERCA2a, calcium-regulated proteins DHPR and Cav1.2 (a), phosphorylation of CaMKII and CREB (b), and the ERS-associated regulatory proteins (c) detected by Western Blotting; ^∗^*p* < 0.05.

The overactivation of ERS can cause oxidative stress damage and induce sarcoplasmic reticulum dysfunction of Ca^2+^ uptake and releasing, furthermore, leading to intracellular Ca^2+^ overload, thereby causing cardiomyocyte apoptosis and affecting myocardial contractile and diastolic function. SERCA2a is one of essential proteins for maintaining cellular Ca^2+^ homeostasis. In this study, SERCA2a was reduced in HF rats (Figure 3(a)), which weakens the SERCA activity and Ca^2+^ transport disorder, leading to inhibition of calcium regulatory proteins, DHPR and Cav1.2 (Figure 3(a)). Calcium overload induces Ca^2+^ binding to CaMKII in order to promote phosphorylation of CaMKII and CREB (Figure 3(b)); simultaneously, the expressions of ERS regulatory proteins, GRP78 and Caspase-12, were both increased in HF rats (Figure 3(c)), implying that ERS can cause SERCA2a degradation and cardiac diastolic dysfunction. As U50488H was administered, SERCA2a was upregulated, the calcium-regulated proteins were activated, leading to CaMKII inhibition and CREB phosphorylation; meanwhile, the expression of ERS regulatory proteins showing ERS injury was also alleviated, suggesting that *κ*-OR agonist can inhibit ERS through activating Ca^2+^-SERCA2a in HF rats.

### 3.4. *κ*-OR Agonist Activates Nrf2/HO-1 Pathway in HF Rats

**Figure 4:**
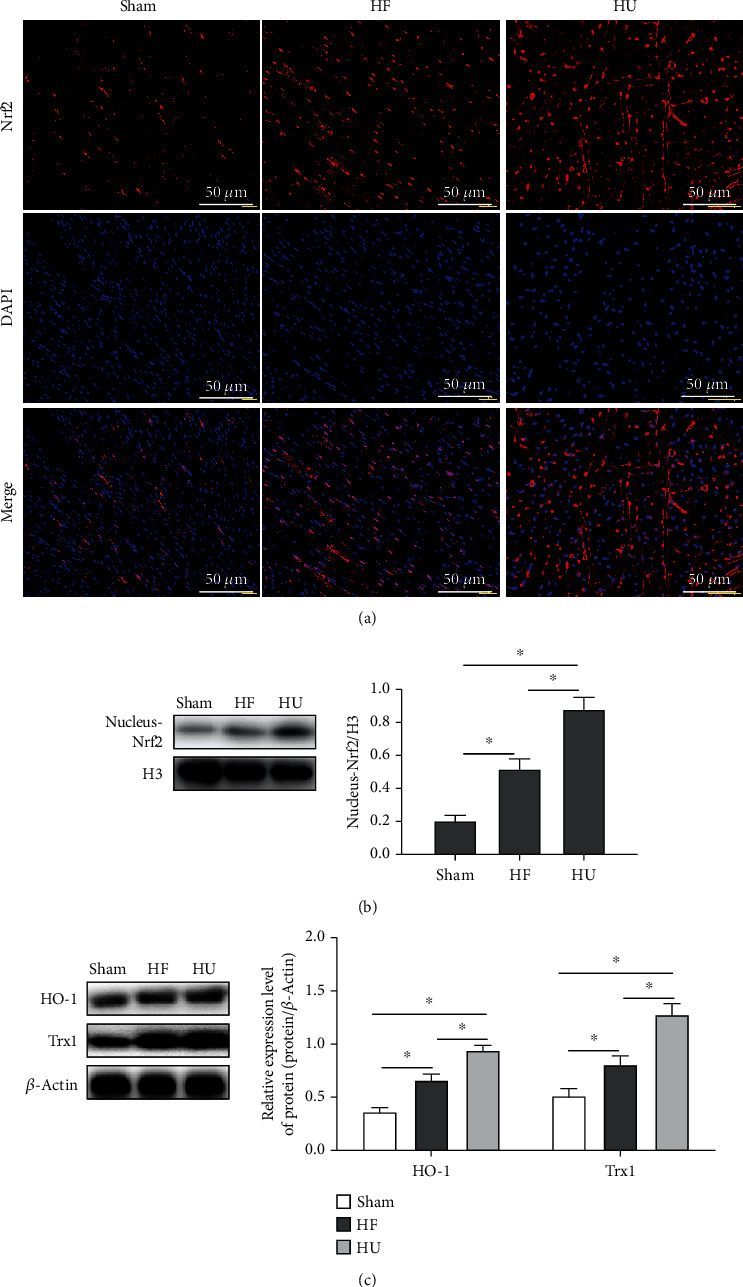
*κ*-OR agonist activates Nrf2/HO-1 pathway in HF rats. (a) The expression of Nrf2 in rats' myocardial nucleus and cytoplasm was detected by IF (scale bar = 50 *μ*m). (b) The expression levels of Nrf2 and its downstream proteins (HO-1 and Trx1) were analyzed by Western Blotting; ^∗^*p* < 0.05.

The balance of the oxidative-antioxidant in ER plays an important role in inhibiting the ER oxidative stress response of cardiomyocytes. Nrf2, an essential regulator in maintaining oxidative-antioxidant balance, is transferred into the nucleus combined with ARE as HF occurs (Figures 4(a) and 4(b), *P* < 0.05); as a result, the release of its downstream antioxidant molecules, HO-1 and Trx1, was stimulated simultaneously (Figure 4(c), *P* < 0.05). U50488H treatment could significantly increase the level of Nrf2 in nucleus (Figures 4(a) and 4(b)). Furthermore, U50488H could also upregulate the expression of HO-1 and Trx1. These results demonstrated that U50488H may activate the Nrf2/HO-1 pathway in HF rats.

### 3.5. CaMKII Agonist Blocks the Protective Effects of *κ*-OR Agonist on Myocardium in HF Rats

**Figure 5:**
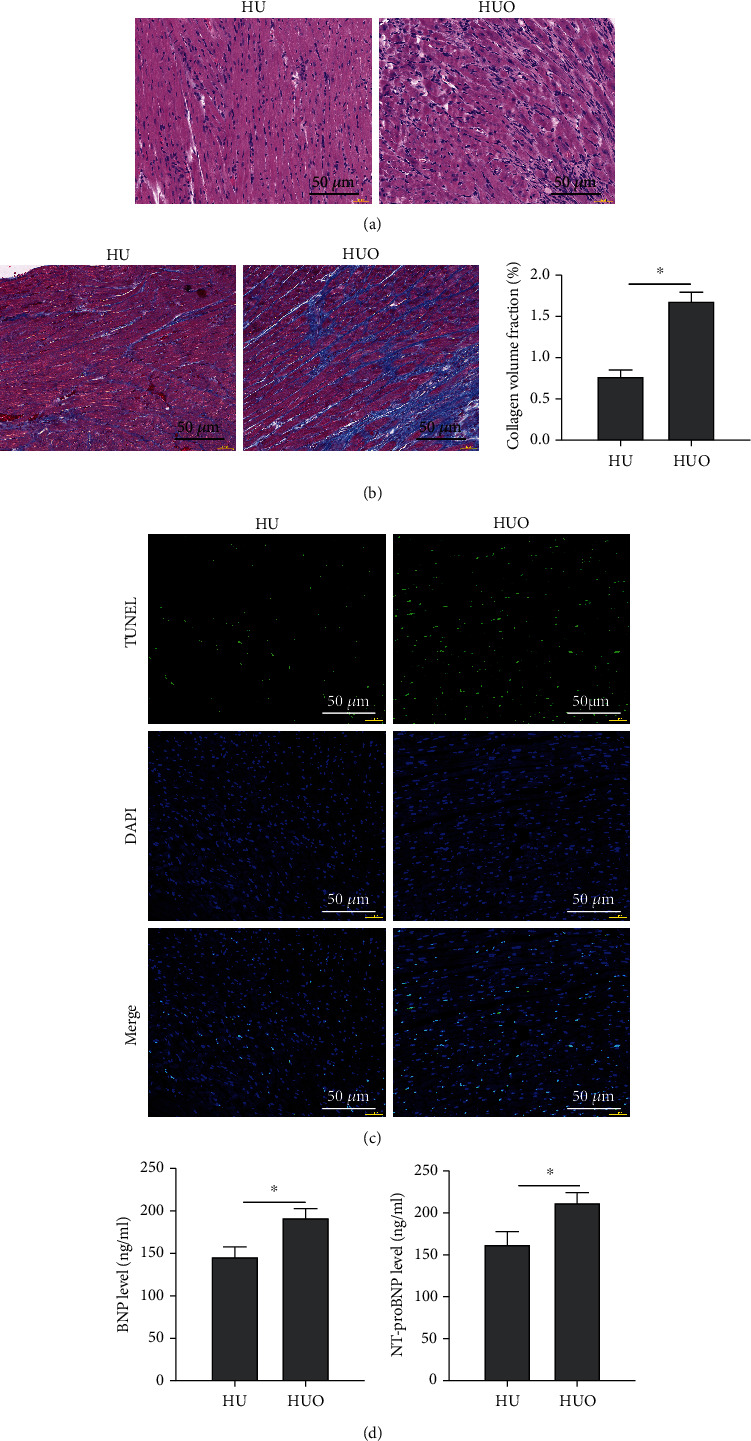
CaMKII agonist blocks the protective effects of *κ*-OR agonist on myocardium in HF rats. (a) Pathological changes in myocardial tissues observed by HE staining (scale bar = 50 *μ*m). (b) The tissue fibrosis was examined by Masson staining (scale bar = 50 *μ*m). (c) The myocardial apoptosis rate was detected by TUNEL staining (scale bar = 50 *μ*m). (d) The natriuretic peptide contents were determined by ELISA; ^∗^*p* < 0.05.

To explore the mechanism of action of *κ*-OR agonists on calcium channels, a specific CaMKII agonist, oleic acid, was administered before U50488H treatment. As shown in Figure 5, the protective effects of *κ*-OR agonists on myocardium in HF rats were suppressed. As a result, distinct myocardial injury (Figure 5(a)) and worsened fibrosis (Figure 5(b)) in rats were observed; increased apoptosis rate (Figure 5(c)) and reduced levels of BNP and NT-proBNP were also demonstrated in the HUO group (Figure 5(d)).

### 3.6. Nrf2 Antagonist Blocks the *κ*-OR-Activated Ca^2+^-SERCA2a

**Figure 6:**
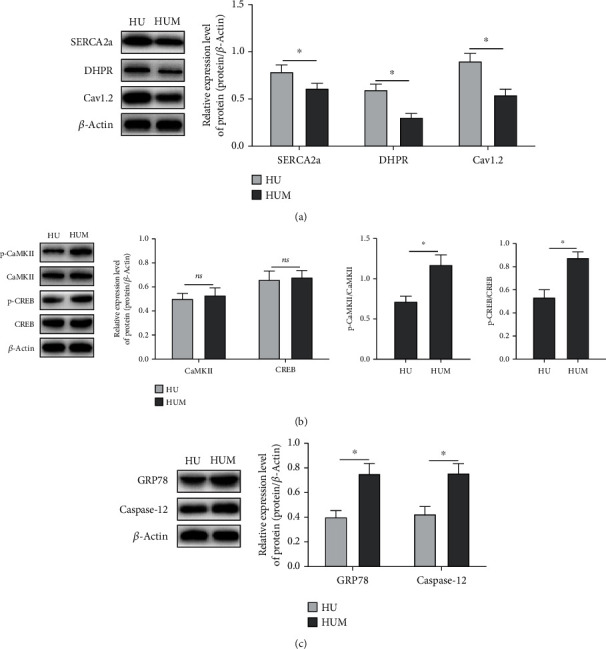
Nrf2 antagonist blocks the *κ*-OR-activated Ca^2+^-SERCA2a. The expression of SERCA2a, calcium-regulated proteins DHPR and Cav1.2 (a), phosphorylation of CaMKII and CREB (b), and the ERS-associated regulatory proteins (c) were detected by Western Blotting; ^∗^*p* < 0.05.

To further explore the regulatory mechanism of Nrf2/HO-1 on Ca^2+^/CaMKII/CREB, the Nrf2 antagonist, ML385, was administered before U50488H treatment. It was observed that Ca^2+^ overload could not be inhibited in HF rats. As shown in Figure 6, the expression levels of SERCA2a (Figure 6(a)) and calcium regulatory proteins (Figure 6(b)) were decreased in the HUM group; thus, the phosphorylation of CaMKII and CREB were promoted. It was also found that the Nrf2 antagonist can improve the expressions of ERS regulatory proteins (Figure 6(c)). These results strongly implied that the protective effects of *κ*-OR agonist on myocardium in HF rats is dependent on activating the Nrf2/HO-1 pathway and regulating Ca^2+^-SERCA2a.

## 4. Discussion

HF is the end-stage manifestation of many cardiovascular diseases [30]. HF is characterized by weakened myocardial contractility and/or diastolic dysfunction and reduced cardiac output, which cannot satisfy the demands of tissue and cell metabolisms [1]. As reflux of venous blood blocked, hemodynamic and body fluids could be changed, leading to a series of symptoms [31]. Epidemiological data shows that the 5-year survival rate of HF patients is similar to that of patients with malignant tumor, and its mortality accounts for 40% of the total mortality caused by many cardiovascular diseases [32]. Abnormal calcium regulation of the sarcoplasmic reticulum in cardiomyocytes is one of the main causes of HF [33]. The damage and reduction of myocardial tissue is one of the key factors of HF. Therefore, it is particularly important to regulate Ca^2+^ level and maintain the stability of cardiomyocytes of HF patients [34]. In this study, the HF rat models were established and administrated with *κ*-OR agonist U50488H. The results showed that U50488H can improve the cardiac functions of HF rats and relieve the myocardial injury. The detailed mechanism is highly correlated to ERS inhibition, Ca^2+^-SERCA2a regulation, and activation of the Nrf2/HO-1 signaling pathway.

*κ*-ORs are widely distributed on the surface of cardiomyocytes and have clear regulatory effects on the function of the cardiopulmonary systems [21]. The area of myocardial infarction and the occurrence of arrhythmia caused by ischemia-reperfusion injury can be reduced through activating *κ*-OR, leading to the reduction of myocardial cells apoptosis and myocardial protection [35]. U50488H is a highly selective *κ*-OR agonist [36]; its specificity for binding and activating *κ*-OR is 1,300 times than the *μ*-ORs, and 12,000 times than the *δ*-ORs. In this study, HF rat models with U50488H treatment demonstrated the improvement on the contractile and diastolic function of the heart, the reduction of myocardial injury, the alleviation of fibrosis, and the reduced cardiomyocyte apoptosis rate. Moreover, the ERS-caused oxidative stress injury was inhibited by U50488H treatment, demonstrating that U50488H could regulate calcium channels to protect myocardium.

Abnormal calcium regulation of myocardial sarcoplasmic reticulum is one of the main factors of HF [37]. The calcium cycle mainly includes the calcium release and recapture in sarcoplasmic reticulum (SR) [37]. Ca^2+^ in the cytoplasm of cardiomyocytes is removed mainly through the reuptake of Ca^2+^ back to SR by SERCA2a after the cardiac excitation-contraction coupling (ECC) process finished. The expression level of SERCA2a is decreased when HF occurs, suggesting that the intracellular calcium overload is caused by Ca^2+^ influx, and Ca^2+^ binds to CaMKII, triggering the phosphorylation reaction of CaMKII where CREB could be phosphorylated by the activated CaMKII. CREB, combined with the specific sequence of cyclophosphadenosine response elements on the target gene, recruit RNA polymerase II to form a transcription complex, thereby regulating the transcription of the target gene. U50488H can upregulate the expression of SERCA2a in HF rats, then activating the expression of calcium-regulated proteins, furthermore, inhibiting the phosphorylation of CaMKII and CREB [13, 14]. Interestingly, the regulatory effect of U50488H on Ca^2+^-SERCA2a was blocked and the protective effect of U50488H on the myocardium was also inhibited, as administration of CaMKII-specific agonist (Oleic Acid), suggesting that U50488H may improve the protection of myocardial calcium and reduce the injury of myocardial tissue by activating Ca^2+^-SERCA2a.

Abnormal expressions of GRP78 and caspase-12, affecting the completion of protein-folding functions, can cause the occurrence of ERS, indicating the steady state of Ca^2+^-SERCA2a in myocardial cells and the homeostasis of oxidative-antioxidant system are destroyed [38]. Nrf2, as the core transcription factor of antioxidative injury, may aggravate cell injury and cause cell dysfunction in the event of activation failure or loss, leading to apoptosis, necrosis, or pyrolysis. Accumulated evidence has proved that the loss of Nrf2 contents can increase myocardial oxidative stress and apoptosis [39], resulting in cardiac dysfunction, while enhancing the expression level of Nrf2 can improve left ventricular function and reduce myocardial hypertrophy in HF rats [40–42]. In this study, Nrf2 undergoes nuclear translocation with the induction of ERS, exhibiting the increased expression level of Nrf2 in the nucleus. It is reported that Nrf2 does not immediately initiate the transcription process unless binding to another transcriptional coactivator, CREB. This is in agreement with the results of enhanced phosphorylation of CaMKII and CREB in HF rats in this study. Interestingly, the regulation of U50488H on the Ca^2+^-SERCA2a homeostasis was inhibited with the application of Nrf2 antagonist, demonstrating that the protective effect of U50488H on the myocardium in HF rats is dependent on activating the Nrf2/HO-1 pathway to regulate Ca^2+^-SERCA2a and to relieve the ERS response in myocardial cells.

Therefore, we demonstrated that U50488H, *κ*-OR agonists, can improve ERS in cardiomyocytes and relieve myocardial injury in HF rats through activating the Nrf2/HO-1 pathway and regulating Ca^2+^-SERCA2a to inhibit Ca^2+^ influx. However, there is still limitation. The effect of Nrf2 activation on the heart is related to the effect of oxidative stress; however, the molecular mechanism that ROS regulating the expression and activation of Nrf2 is not fully demonstrated in this study. We will investigate the molecular mechanism in further studies.

## Data Availability

The datasets used and analyzed during the current study are available from the corresponding author upon reasonable request.
